# Olefin metathesis catalysts embedded in β-barrel proteins: creating artificial metalloproteins for olefin metathesis

**DOI:** 10.3762/bjoc.14.265

**Published:** 2018-11-19

**Authors:** Daniel F Sauer, Johannes Schiffels, Takashi Hayashi, Ulrich Schwaneberg, Jun Okuda

**Affiliations:** 1 Institute of Inorganic Chemistry, RWTH Aachen University, Landoltweg 1, 52074 Aachen, Germany; 2 Institute of Biotechnology, RWTH Aachen University, Worringerweg 3, 52074 Aachen, Germany; 3 Department of Applied Chemistry, Graduate School of Engineering, Osaka University, 2-1 Yamadaoka, Suita 565-0871, Japan

**Keywords:** artificial metalloprotein, β-barrel protein, metathease, olefin metathesis, ruthenium

## Abstract

This review summarizes the recent progress of Grubbs–Hoveyda (GH) type olefin metathesis catalysts incorporated into the robust fold of β-barrel proteins. Anchoring strategies are discussed and challenges and opportunities in this emerging field are shown from simple small-molecule transformations over ring-opening metathesis polymerizations to in vivo olefin metathesis.

## Introduction

Olefin metathesis constitutes the rearrangement of C=C double bonds in the presence of transition metal catalysts based on V, Mo, W, Re, Ru, and Os together with alkylating co-catalysts. This transformation is widely used in organic synthesis as well as in polymerization of various unsaturated monomers [1]. According to the Chauvin mechanism, the catalytically active species are Schrock-type carbenes or alkylidenes [2]. Olefin metathesis greatly profited from the isolation of structurally well-defined metal alkylidene complexes [3–4]. The best studied and most commonly employed catalysts are based on Mo, W, and Ru [1].

Initially, these complexes were considered to be sensitive towards air and moisture. Nevertheless, adding Ru, Os and Ir salts to an aqueous solution or emulsion of a norbornene derivative led to ring-opening metathesis polymerization to give the corresponding polymer [5–6]. Through modification of the first coordination sphere by adding an *N*-heterocyclic carbene (NHC) ligand and a chelating styrene to the so-called Grubbs 1st generation catalyst, the relatively air- and moisture-stable Grubbs–Hoveyda type (GH-type) catalysts were obtained [7]. These catalysts do not only show stability towards moisture, but can also be directly used in water, allowing to perform olefin metathesis reactions in aqueous solutions [8–9].

Olefin metathesis is not known in biological systems and therefore can be regarded as bio-orthogonal. The group of Davis utilized the olefin metathesis reaction to perform post-expressional protein modifications [10–12]. For example, a single cysteine mutant of subtilisin from *Bacilus lentus* (SBL-S156C) was modified via sulfide bond formation with allyl cysteine displaying an allyl function on the protein surface. This allyl group was modified with a GH-type catalyst and carbohydrate or small polyethylene glycol (PEG) groups were attached [11]. As another strategy to modify a protein surface with olefin metathesis, Isarov and Pokorski introduced a Grubbs 3rd generation catalyst on the surface of lysozyme and performed ring-opening metathesis polymerization (ROMP) on the protein surface employing a PEGylated norbornene derivative as substrate [13]. This led to proteins modified with PEG chains. These two examples illustrate the potential applications of olefin metathesis in protein modification. Further applications would be the implementation of olefin metathesis into natural metabolic pathways to allow synthesis of fine chemicals [14]. Also, a targeted reaction in a certain environment within a living cell with a precise release or activation of the catalyst would enable new ways of drug delivery. The challenge to overcome this regard is the deactivation of the catalyst inside the cells and the transport within organisms without triggering or activating a response of the corresponding target [15]. Additionally, the (kinetic) stability of the catalysts in aqueous solutions needs to be improved for this purpose. For application in organic synthesis in aqueous environments, water solubility is also essential [16–18].

**Scheme 1:**
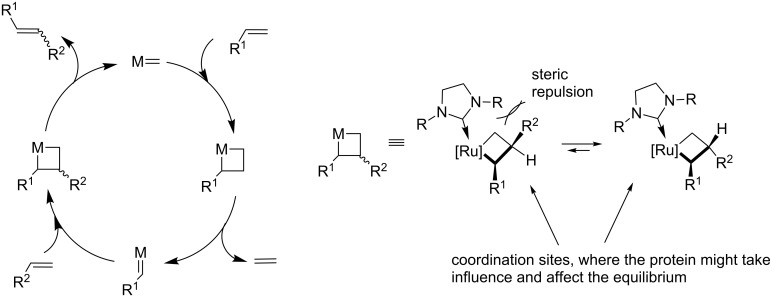
Left: Mechanism of the olefin metathesis reaction postulated by Chauvin [2]. Right: Potential influence of the protein as second coordination sphere in the transition state that lead to different metathesis products.

A promising approach is the embedment of the GH-type catalyst into well-defined protein scaffolds [19]. The combination of an engineered protein with a synthetic metal catalyst leads to artificial metalloproteins [20–23]. In the case of a metathesis catalyst, so-called artificial metatheases are obtained, which could open new areas of biological applications [19]. The protein as second coordination sphere might take influence on the formation of the metallacyclobutane that was initially postulated by Chauvin [2]. The formation of the *E* or the *Z* product is dependent on the orientation of the R groups in this step of the catalytic cycle (Scheme 1).

**Scheme 2:**
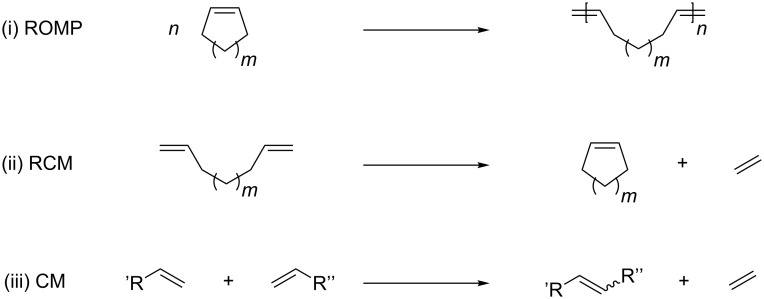
(i) Ring-opening metathesis polymerization (ROMP), (ii) ring-closing metathesis (RCM) and (iii) cross metathesis (CM).

In this short review, we focus on the status of embedding the GH-type catalyst into β-barrel proteins and show their application in various reactions using benchmark substrates. These transformations include all three fundamental olefin metathesis reactions: ring-opening metathesis polymerization (ROMP), ring-closing metathesis (RCM) as well as cross metathesis (CM) (Scheme 2).

## Review

### Artificial metatheases – anchoring approaches

Metalloproteins that contain one or more metal ions such as Mg, Ca, Mn, Fe, Ni, Co, Cu, Zn etc. within a protein are abundant in nature [24]. As metalloenzymes, these metalloproteins are capable of catalyzing various important reactions in biosynthesis and key steps in cellular energy metabolism. The embedded metal ion mainly acts as a Lewis acid catalyst or redox catalyst. Various metalloenzymes have been applied in laboratory-scale reactions and a few metalloenzymes such as nitrile hydratase (cobalt(III) in the active site) for the production of acrylamide have found application in industry [25]. Notably, however, the reaction scope of natural enzymes is quite limited. Apart from engineering natural enzymes, the approach of connecting abiotic co-factors (such as organometallic complexes) to natural or re-engineered protein scaffolds offers an attractive combination of both, broad reaction scope of chemical transformations as well as control of selectivity and specificity as found in natural enzymes. These so-called artificial metalloproteins or metalloenzymes offer two ways of fine-tuning activity and selectivity: As chemical means, the metal site can be adjusted and fine-tuned through modification of the ligands surrounding the metal. As biotechnological means, the protein cavity acting as second coordination sphere can be optimized to tune specificity as well as stereo- and regioselectivity. The extensive literature of artificial metalloproteins has been summarized in various comprehensive reviews [20–22].

**Figure 1:**
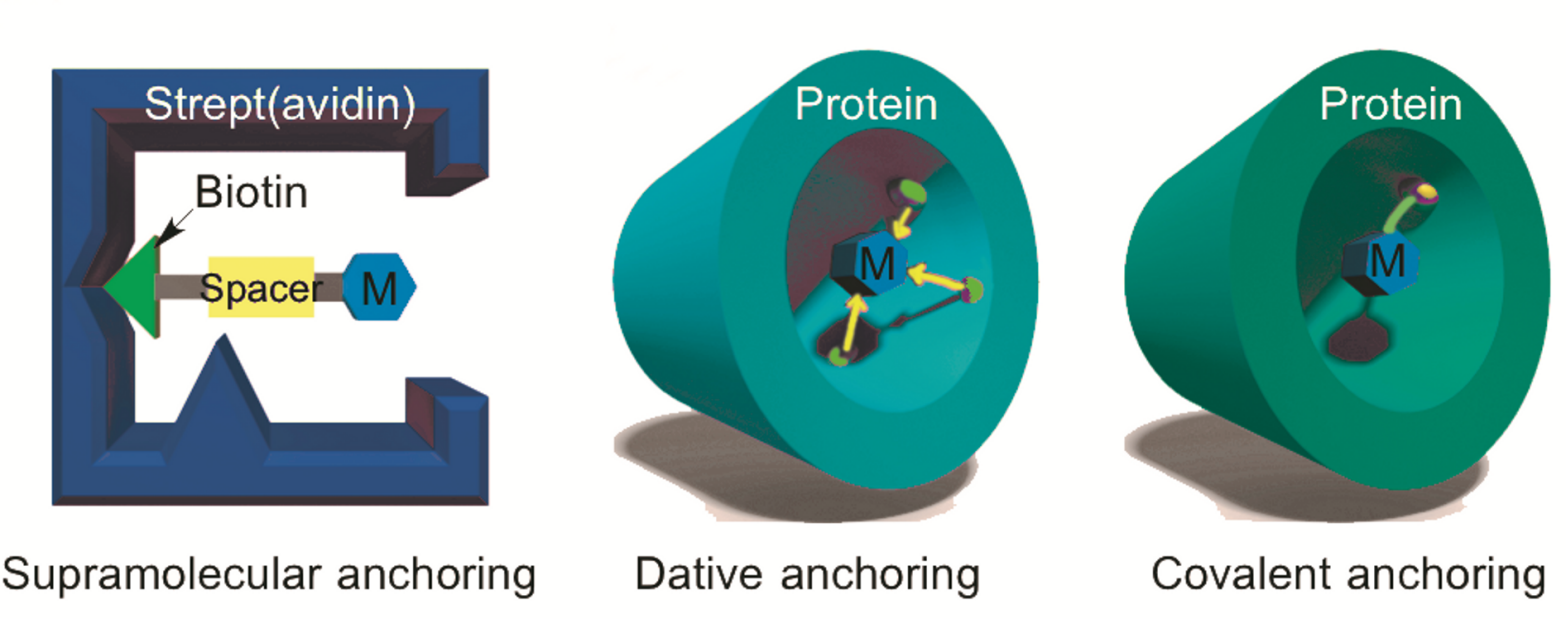
Common anchoring strategies for metal-complex or metal ion incorporation into protein scaffolds.

One of the challenges to overcome in the construction of artificial metalloproteins is to find a method to incorporate a synthetic metal complex into a protein scaffold [26]. The common strategies are shown in Figure 1.

In Figure 1, the three commonly utilized methods to incorporate a synthetic cofactor are shown. Strategies utilized are supramolecular, dative and covalent anchoring. Supramolecular anchoring was pioneered by Wilson and Whitesides in 1978 [27]. They made use of the high affinity of (strept)avidin (Sav) to biotin that represents one of the strongest supramolecular interactions found in nature with a dissociation constant of approximately *K*_d_ ≈ 10^−15^ M [28]. Initially, an achiral Wilkinson-type catalyst was attached to perform hydrogenation [27]. Nowadays, a broad variety of artificial metalloproteins based on this technology has been established [20,29]. Dative anchoring offers the possibility to liberate the active site from the protein easier as compared to supramolecular anchoring. However, the design of catalysts capable of undergoing dative anchoring is usually based on interactions of inhibitors with the active site of the protein. This makes the catalyst design challenging and the application is limited. Covalent anchoring of an organometallic complex offers the precise positioning of a catalyst within a protein scaffold. Formation of the covalent bond between cofactor and protein ensures an irreversible binding of the active site (i.e., the metal complex). This approach is highly versatile, because it is not necessary to have or to design interactions that are required for non-covalent anchoring, e.g., supramolecular or dative anchoring.

All three anchoring approaches – supramolecular, dative and covalent – have been utilized to construct artificial metalloproteins capable of catalyzing olefin metathesis reactions [19]. To date, eight artificial metatheases have been reported. Among them, β-barrel proteins play a central role as protein scaffolds.

### β-Barrel proteins

Proteins are constructed from two major secondary structural elements, namely α-helices and β-sheets. Notably, the latter are generally regarded to be more rigid than disordered or α-helix structures [30–31]. β-Barrels are structural motifs found in numerous proteins in which (mostly) antiparallel β-strands twist and coil to form closed, quasi-cylindrical structures held together by a network of hydrogen bonds [32]. Characterized by an amphiphilic nature with either hydrophobic “barrel” interiors and hydrophilic surfaces (as in globulins, carriers of hydrophobic molecules and fluorescent proteins) or hydrophilic cores and hydrophobic surfaces (as in membrane-bound β-barrels like porins and channel proteins), they can be present as minor motifs or even dominate the overall protein structure [33–34].

Small β-barrels such as lipocalins (i.e., transporters of small hydrophobic molecules that play vital roles in many biological processes [35]) or heme-containing nitrophorins/nitrobindins of the all-β-barrel type (involved in NO transport, storage and sensing as well as heme metabolism [36]) usually constitute eight to ten antiparallel β-strands and tightly packed hydrophobic or hydrophilic barrel interiors [37]. Membrane-bound β-barrels are confined to mitochondrial and chloroplast membranes and the outer membranes of Gram-negative bacteria [38]. They constitute up to 24 strands, require sophisticated assembly machineries for membrane integration [39] and are usually “plugged” by hydrophilic loops and helices that either ensure the binding of small molecules, or their (energy-dependent) transport across the outer membrane. TIM-barrels (named after triosephosphate isomerase, TIM), in turn, contain both α- and β-structures, i.e., a β-barrel structure (eight strands) enclosed by a series of eight α- helices. The TIM-barrel represents a very common – yet evolutionarily diverse – protein structure [40].

While following very similar structural patterns, β-barrel and TIM-barrel proteins occupy a tremendous sequence space and are highly versatile in terms of metabolic functions, binding properties, transport and catalytic activities. The compact barrel structure can be regarded as a prototype of stable protein scaffolds/motifs exhibiting stabilities against a wide range of external influences including high salt concentrations, high temperatures and organic solvents [41–45]. These properties make them excellent scaffolds for the construction of artificial metalloenzymes, which is achieved by removing the native cofactors or the cork/plug domains to reveal otherwise occupied pockets or pores that can then be loaded with artificial catalysts.

### Artificial metatheases within β-barrel proteins

#### (Strept)Avidin

**Scheme 3:**
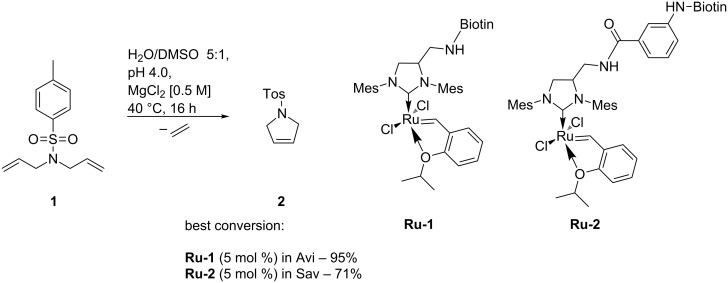
Biotinylated GH-type catalysts for conjugation to (strept)avidin and their catalyzed ring-closing metathesis [46].

Artificial metalloproteins for olefin metathesis based on the supramolecular anchoring approach were synthesized by Ward [29]. A GH-type second generation olefin metathesis catalyst was modified at the periphery of an NHC ligand with a biotin moiety [46]. The small β-barrel protein avidin (Avi) or streptavidin (Sav) was incubated with the catalyst to give the artificial metalloprotein. This (strept)avidin-based catalyst was tested in the RCM reaction of *N*,*N*-diallyl-4-toluenesulfonamide (**1**) in aqueous buffer solution [46]. Conversions up to 95% with Avi as a protein scaffold were achieved (catalyst loading of 5 mol %). This was the first example describing olefin metathesis performed within a protein cavity. During this study, already a hint at the importance of the spacer length became apparent. A short spacer between the GH-type catalyst (**Ru-1**) and the biotin moiety did not lead to a successful conversion of the substrate. Elongation of the spacer (**Ru-2**) and therefore moving the active site slightly out of the protein cavity led to improved conversion (Scheme 3) [46].

**Scheme 4:**
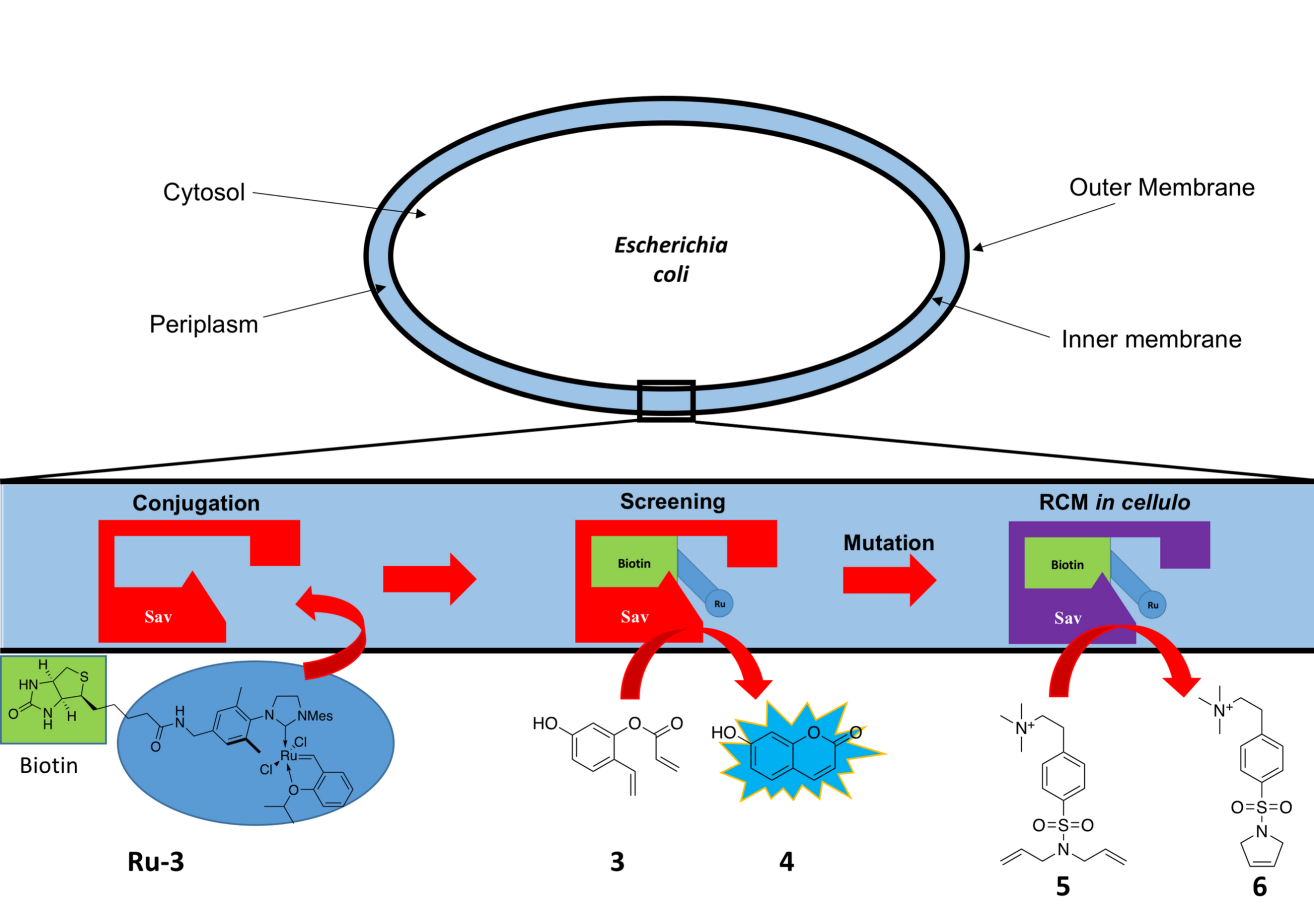
Whole-cell artificial metatheases designed by Ward et al. [47].

The combination of the GH-type catalyst and (strept)avidin was further developed in a system that performs RCM reactions within a whole cell [47–48]. The scaffold protein Sav was produced into the periplasm of *Escherichia coli* (*E. coli*) [47]. The recombinant cells were incubated with a biotinylated GH-type catalyst **Ru-3** that reaches the target protein via diffusion through the outer membrane (Scheme 4). Characterization of this whole-cell system included ICP analysis. Whole-cells containing Sav showed an approximately three-fold increase in ruthenium content as compared to cells lacking the Sav variant (80,000 Ru atoms per cell and 29,000 Ru atoms per cell, respectively) [47].

This system was subjected to directed evolution. The twenty amino acid positions closest to the active site were saturated, and the best mutant formed the starting variant for the next iterative round. As screening substrate, the pre-fluorescent styrene derivative **3** was used. Following RCM, the fluorescent molecule umbelliferone (**4**) was generated. In total, five rounds of directed evolution were performed, yielding the mutant Sav_K121R_N49K_A119G_T114Q_V47A (Sav_Mut) [47].

As a rescreening, the RCM reaction of a water-soluble, charged diallylamine **5** was performed. Cells harboring the Sav_WT, Sav_Mut and no Sav were tested. Whole-cell Sav_WT and Sav_Mut reached both a turnover number per cell TON(per cell) of about 300,000. Cells without Sav reached TON(per cell) ≈ 20,000. The small difference between Sav_WT and the mutant Sav_Mut is explained by electrostatic repulsion of the positively charged substrate and the arginine at position 121. Another round of site-saturation mutagenesis yielded the variant Sav_R121L_N49K_A119G_T114Q_V47A (Sav_Mut2), which exhibited an improved activity of TON(per cell) ≈ 500,000 compared to Sav_WT [47]. This is the first example of a whole-cell metathesis biohybrid catalyst, opening up new possibilities to utilize olefins in biological systems in the context of artificial metabolism [14].

#### Nitrobindin

Nitrobindin (NB) is a small, soluble β-barrel protein with a molecular weight of 19 kDa [49]. NB wild-type has 10 β-strands and contains a heme as a prosthetic group [49]. Upon modification of the axial histidine that coordinates the heme, the robust β-barrel structure with a relatively small cavity is retained [50].

Further mutations within the cavity of NB provide a hydrophobic cavity. Several studies reported on the utilization of NB as scaffold for incorporated metal complexes, including the work of Hayashi et al. capitalizing on the polymerization of phenylacetylene [50–51], the Diels–Alder reaction [52–53], and hydrogen evolution [54]. Further, Lewis et al*.* employed the NB scaffold for epoxidation of styrene and other olefins [55]. In all studies, the catalyst incorporated into the NB scaffold showed increased activity as compared to the protein-free catalyst under similar conditions.

**Scheme 5:**
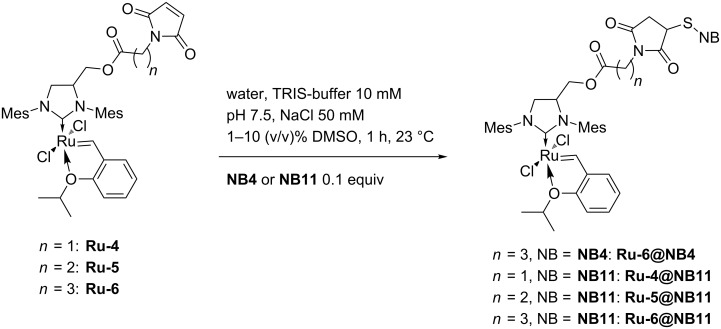
Coupling of GH-type catalysts **Ru-4/5/6** to **NB4** or **NB11**.

Engineered variants of NB were used to construct artificial metatheases [56]. The cavity of NB was enlarged by introducing five mutations compared to the NB wild-type. Two histidines were substituted by leucine or alanine. Furthermore, a cysteine was introduced allowing covalent anchoring, and the two methionines inside the cavity were substituted by leucines. This yielded the two mutants **NB4** (leucine for histidine; mutations in comparison to NB wild-type: M75L/H76L/Q96C/M148L/H158L) and **NB11** (alanine for histidine; mutations in comparison to NB wild-type: M75L/H76L/Q96C/M148L/H158A) [56]. Notably, the introduced mutations further affected the cavity size of the proteins. **NB4** has a cavity volume of 855 Å^3^ and **NB11** has an enlarged volume of 1161 Å^3^ [52,56]. These two mutants were tested for the construction of artificial metatheases. As catalyst, GH-type catalysts with different spacer lengths were investigated, including methylene (**Ru-4**), ethylene (**Ru-5**) to a propylene (**Ru-6**) spacers [56]. Thereby, it was aimed to locate the active center properly within the protein cavity. The challenge in the conjugation of the GH-type catalyst into narrow protein cavities is to overcome the space demand of the bulky NHC ligand. The conjugation was performed via maleimide-thiol “click” reaction under slightly basic (pH 7.5) conditions. Within the small cavity of **NB4**, only the GH-type catalyst **Ru-6** with the longest spacer was able to undergo conjugation; however, the conjugational yield was very low (25%). Within the bigger cavity of **NB11**, all three catalysts **Ru-4/5/6** were able to undergo conjugation, and gradually increasing conjugation yields by elongation of the spacer was observed (from 29% for **Ru-4** up to 89% for **Ru-6**; Scheme 5) [56].

These artificial metalloproteins were purified and characterized by different analytical methods [56]. Structural integrity of the β-barrel fold was confirmed by CD spectroscopy. ICP–OES was used to determine the metal content. A little less than one metal center per protein molecule was found to be present. Additional absorption bands in the UV–vis spectra around λ = 380 nm indicated the presence of the GH-type catalyst. Finally, the peak for the biohybrid conjugate was observed in ESI–TOF–MS suggesting successful covalent anchoring.

Beside ring-closing metathesis (RCM) of 2,2-diallylpropane-1,3-diol to yield the corresponding cyclopentane derivative, the synthesized biohybrid catalysts were tested in the ring-opening metathesis polymerization of a 7-oxanorbornene derivative **7** (Table 1) [56].

**Table 1:** Ring-opening metathesis polymerization (ROMP) of oxanorbornene **7** catalyzed by artificial metatheases based on NB.

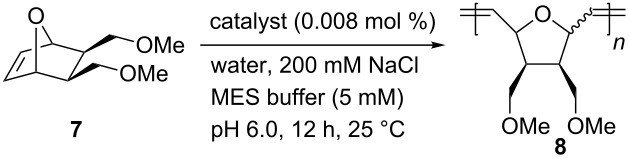				

Entry	Catalyst	Conversion^a^ [%]	*cis*/*trans*^a^	TON

1^b,c^	**Ru-4/5/6**	<5	n.d.	n.d.
2	**Ru-6@NB4**	10	40:60	1100
3	**Ru-4@NB11**	<5	n.d.	n.d.
4	**Ru-5@NB11**	18	43:57	2000
5	**Ru-6@NB11**	78	43:57	9700

^a^Determined by ^1^H NMR spectroscopy in CDCl_3_; ^b^containing 10% (v/v) THF; ^c^catalyst loading: 0.01 mol %.

With a catalyst loading as low as 0.01 mol %, no activity of the protein-free catalysts Ru-**4/5/6** was detected (Table 1, entry 1) [56]. In turn, the catalysts immobilized within the protein cavity showed activity. Within the small cavity of **NB4**, moderate conversions up to 10% were obtained, and activity was only observed when **Ru-6** (longest spacer) was incorporated (Table 1, entry 2) [56]. By contrast, within the larger cavity of **NB11**, all catalysts **Ru-4/5/6** showed activity (Table 1, entries 3–5). Again, **Ru-6** (longest spacer) was most effective among the catalysts, and up to 78% conversion (corresponds to a TON = 9700; Table 1, entry 5) were achieved with the corresponding **Ru-6@NB11** [56]. The corresponding polymer had a molecular weight of *M*_n_ = 180,000 g/mol and a narrow molecular weight distribution (PDI = 1.05), suggesting the living nature of the ROMP even within the protein scaffold. Neither regioselectivity (*cis*/*trans*) nor tacticity were affected [56].

#### The transmembrane protein FhuA

The β-barrel proteins introduced for the construction of artificial metatheases up to this point are relatively small and soluble proteins. As introduced vide supra, membrane-spanning porins and transporters of the all-β-barrel type, which are found in cellular outer membranes, constitute substantially larger “barrel” interiors and were thus utilized as scaffolds to house bulky GH-type catalysts.

**Scheme 6:**
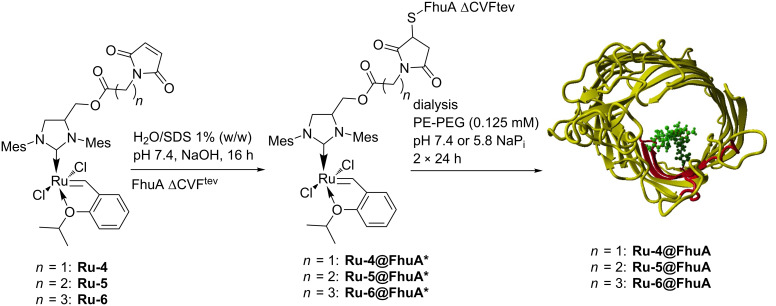
Anchoring and refolding of GH-type catalysts **Ru-4/5/6** to FhuA.

*Ferric hydroxamate uptake protein component A* (FhuA) is naturally located in the outer membrane of *E. coli*, where it is involved in cellular iron import. It has a robust β-barrel structure consisting of 22 antiparallel β-strands [57]. By genetic engineering, Braun and co-workers modified this transporter and removed the cork domain that is responsible for the iron transport [58]. This generated an “empty” barrel offering sufficient space to incorporate bulky organometallic catalysts. The variant lacking the cork domain is termed FhuA Δ1-159 (amino acids from 1 to 159 are deleted compared to the wild-type protein). For covalent anchoring, a cysteine residue was introduced at position 545 [59]. This position is suggested to be in a conformationally stable environment within the β-barrel structure. Additionally, mutation N548V was introduced to enable access of the metal catalyst to position C545. Furthermore, E501 was substituted by phenylalanine to prevent coordination of the Glu side chain to the metal site and deactivation of the catalyst. Two specific TEV (Tobacco Etch Virus protease) cleavage sites were further introduced into loops 7 and 8 to facilitate MALDI–TOF–MS analysis. The final mutant utilized for the construction of artificial metatheases is termed FhuA Δ1-159_C545_V548_F501_tev (FhuA ΔCVF^tev^) [59]. Conjugation was performed with GH-type catalysts **Ru-4/5/6** in the presence of SDS (Scheme 6).

Utilization of SDS as detergent resulted in partial denaturation of the FhuA – called unfolded FhuA – and facilitates the access of the GH-type catalysts to the cysteine C545 [59]. The resulting biohybrid catalysts Ru-4/5/6@FhuA* were washed repeatedly to remove unbound catalyst. The protein structure was restored (“renaturation”) leading to the refolded biohybrid catalysts **Ru-4/5/6@FhuA** (Scheme 6) which were tested in the ROMP of oxanorbornene **7** (Table 2) [59–60].

**Table 2:** ROMP of substrate **7** catalyzed by **Ru-4/5/6@FhuA**.

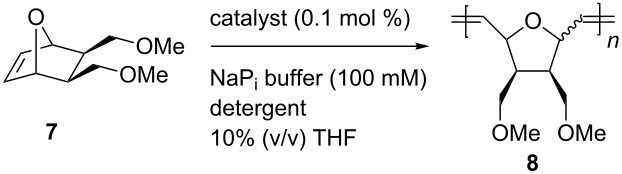						

Entry^a^	Catalyst	Detergent	pH	Conv.^b^ [%]	TON	*cis*/*trans*^b^

1	Ru-4@FhuA*	SDS^c^	7.4	90	900	60/40
2	Ru-4@FhuA*	SDS^c^	5.8	99	990	61/39
3	Ru-5@FhuA*	SDS^c^	5.8	99	990	60/40
4	Ru-6@FhuA*	SDS^c^	5.8	99	990	60/40
5	**Ru-4@FhuA**	PE-PEG^d^	7.4	7	94	57/43
6	**Ru-4@FhuA**	PE-PEG^d^	5.8	41	555	58/42
7	**Ru-5@FhuA**	PE-PEG^d^	5.8	24	325	56/44
8	**Ru-6@FhuA**	PE-PEG^d^	5.8	37	365	56/44

^a^Conditions: Protein concentrations determined with BCA assay and catalyst loading determined with ThioGlo titration (approx. 0.09 mM); ^b^determined by ^1^H NMR spectroscopy in CDCl_3_; ^c^containing 1% (w/w) SDS; ^d^[PE-PEG] = 0.125 mM.

The biohybrid catalysts Ru-4/5/6@FhuA* in SDS solution showed activities comparable to the protein-free catalyst (Table 2, entries 1–4) [59–60]. Under slightly basic conditions (pH 7.4), 90% conversion was achieved (Table 1, entry 1). Under slightly acidic conditions (pH 5.8), full conversion was observed with the metal complex coupled to the fully unfolded protein (Table 2, entries 2–4) [59–60]. This effect was attributed to the pH and was investigated in detail [61].

After refolding, the activity decreased (Table 2, entries 5–8) [59–60]. This may be related to the steric demand of the refolded β-barrel structure that fully surrounds the metal site. Additionally, the activity of catalyst **Ru-6@FhuA** with the shorter linker increased (Table 2, entry 6 compared to entries 7 and 8) [60]. The restricted movement of the catalyst with shorter spacer within the channel seems advantageous for the turnover. Additionally, a few potentially coordinating residues (glutamic acid and tyrosine) are further away from the active site when the shorter spacer is utilized [60].

### Structural expansions of β-barrel proteins

**Figure 2:**
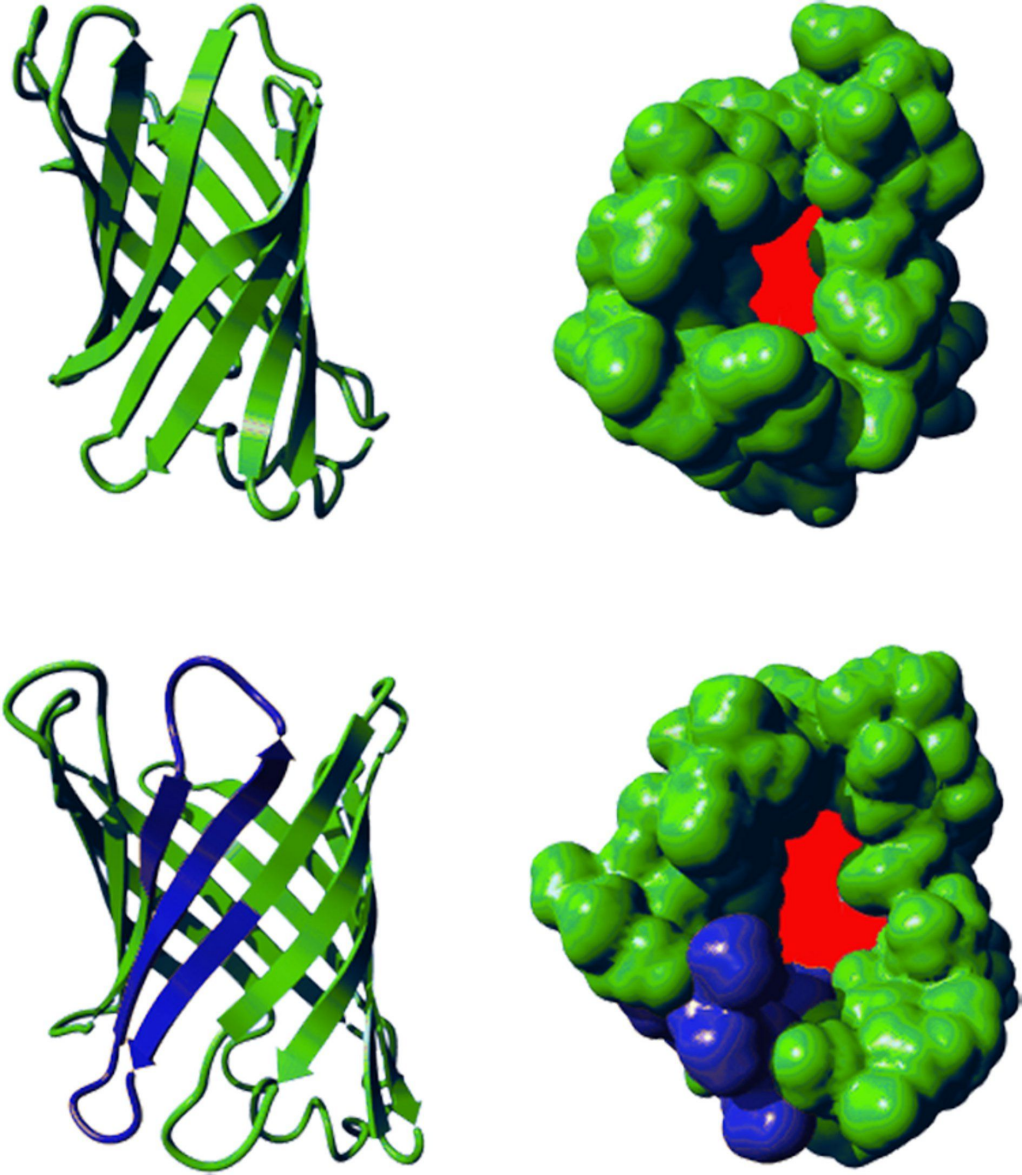
Top: NB4 (PDB 3WJB); bottom: NB4exp. Highlighted in blue are the additional two β-sheets. Highlighted in red is the diameter of the cavity.

Comparing the activities of biohybrid catalysts based on the small β-barrel proteins NB and Sav with the large membrane protein FhuA reveals striking differences. Interestingly, much higher activities were observed when the catalysts were incorporated into the cavities of small β-barrel proteins. For the ROMP reaction, no change in regioselectivity was observed in both proteins. Within FhuA, the activity significantly dropped. This observation suggests that a particular fine-tuning is required to optimally utilize the combination of the metal catalyst with the spacing unit and the protein‘s precise 3D-structure that forms the second coordination sphere of the metal ion. The active site needs to be situated in the protein cavity to sense the protein environment. The cavities of NB and Sav are too small to fully surround the bulky catalysts. Methods have been developed to enlarge the cavity or to introduce additional structural motifs to improve the protein–metal interaction. In case of NB4, two additional β-sheets were introduced to give a variant comprising 12 β-sheets, denoted expanded NB (**NB4exp**) [62]. These two additional β-sheets increased the cavity volume from 855 Å^3^ to 1399 Å^3^ (Figure 2) [62].

**NB4exp** was subjected to conjugation of catalysts **Ru-4/5/6**. Indeed, all catalysts underwent covalent anchoring with high conjugational yield (confirmed via ICP–OES and ESIMS) [62]. Upon catalysis, **Ru-5@NB4exp** as well as **Ru-6@NB4exp** showed high activity in the ROMP of norbornene **7** with TONs up to 10,000. For the catalyst **Ru-4** with the short linker, the activity of **Ru-4@NB4exp** dropped to TON = 3,000, even though the conjugation was almost quantitative [62]. However, this “influence” on the activity could not be transferred to the regio- and stereoselectivity of the polymer microstructure. Apart from ROMP, the artificial metatheases based on **NB4exp** were capable of catalyzing both CM and RCM. This makes **NB4exp** based biohybrid catalysts the first artificial metatheases to catalyze all basic metathesis reactions [62].

For the artificial metathease based on Sav, additional structural motifs – α-helices – were introduced into the loops. These loops are supposed to embed the active site. However, in first ring-closing metathesis reactions, the influence of the newly introduced α-helices was negligible [63].

## Conclusion

In this review, we discussed the combination of GH-type catalysts and β-barrel proteins to construct artificial metatheases. The β-barrel motif offers a robust, well-defined but easily modifiable second coordination sphere. This makes the artificial metatheases applicable in all basic metathesis reactions. The channel provided by β-barrel proteins is a potentially useful feature to immobilize the GH-type complex within the protein. So far, no advantage has been drawn out of this feature. Strategies to enlarge small cavities of small β-barrel proteins likely will lead to more selective artificial metatheases. Directed evolution may open new opportunities for catalyst optimization [64].
